# NEX4EX – A novel exercise device enabling resistive, plyometric and sensorimotor training during deep‐space missions: A case report

**DOI:** 10.1113/EP092721

**Published:** 2025-09-15

**Authors:** Jonas Böcker, Jochen Zange, Markus Gruber, Guillaume Fau, Rainer Rawer, Sören Törholm, Arnaud Runge, Jörn Rittweger

**Affiliations:** ^1^ Institute of Aerospace Medicine, German Aerospace Center Cologne Germany; ^2^ Human Performance Research Centre, Department of Sport Science University of Konstanz Konstanz Germany; ^3^ Space Applications Services Sint‐Stevens‐Woluwe Belgium; ^4^ Novotec Medical GmbH Pforzheim Germany; ^5^ Anybody Technology Aalborg Denmark; ^6^ European Space Agency ESTEC Noordwijk The Netherlands; ^7^ Department of Pediatrics and Adolescent Medicine University Hospital of Cologne Cologne Germany

**Keywords:** case report, countermeasure exercise, plyometric training, resistive training, sensorimotor training

## Abstract

During weightlessness, the human neuro–muscular–skeletal system undergoes maladaptation to the microgravity environment. The European Space Agency (ESA) project NEX4EX, ‘Novel Exercise Hardware for Exploration’, developed an advanced multipurpose exerciser offering resistive (RES), plyometric (PLYO) and sensorimotor (SENSO) exercises. It is the aim of this case report to assess the functionality of the device. NEX4EX offers RES in terms of squats and heel raises, and PYLO in terms of countermovement jumps and hops. RES and PLYO were compared with standard exercises on ground as reference. SENSO were generated by creating disturbances of the body posture by means of random, rapid pulling on a shoulder harness in four directions and by an oscillating platform. For SENSO, the results showed clear postural reflexes in trunk and leg muscles to stabilise upright posture after perturbation stimuli at the shoulders. RES and PLYO were carried out accurately on NEX4EX by the participants, but with reduced loads compared to reference (up to −37% for RES; up to −24% for PLYO). This resulted in reduced muscle activation for RES, whereas the muscle activation stayed comparable for PLYO. A reduced maximum take‐off velocity during PLYO (up to −66%) was shown leading to a reduced jump height (up to −72%). Although some exercises could not be performed with the same intensity with NEX4EX, in general it enabled all intended exercises. The basic functionality of the device was shown, and thus the device showed its potential as an integrative countermeasure device for upcoming deep‐space missions.

## Abstract (highlights)

- **What is the central question of the study?**
  The NEX4EX device was designed for resistive, plyometric and sensorimotor exercises: is the novel device capable of enabling all intended exercises?
- **What is the main finding and its importance?**
  All exercises were feasible using NEX4EX. Resistive and plyometric exercises were possible with reduced loads. The sensorimotor exercises evoked postural reflex activities of trunk and leg muscles. Thus, the device enabled all intended exercises and has great potential for being an adequate countermeasure device for deep‐space missions.

## 1 INTRODUCTION

The lack of mechanical loading in weightlessness causes muscle loss (Narici & de Boer, 2011), bone degeneration (LeBlanc et al., 2007) and reduced neuromuscular performance (Pisot et al., 2016) and deconditions involuntary motor control programs (Shpakov et al., 2013). Maintaining a crew's operational skills requires countermeasures that prevent such maladaptations to ensure the health of the space travellers going on deep‐space missions. Resistive exercise devices already exist on the International Space Station (ISS) (Petersen et al., 2016; Scott et al., 2021). Plyometric exercises are not used on the ISS yet, but showed great potential during bed rest studies (Kramer, Gollhofer et al., 2017; Kramer, Kümmel; Ritzmann et al., 2018). Furthermore, no specific countermeasure device exists to implement sensorimotor exercises during microgravity. Thus, it became the aim to integrate all these aspects in one countermeasure device, NEX4EX (Novel Exercise Hardware for Exploration), capable of performing resistive (RES), plyometric (PLYO) and sensorimotor (SENSO) exercises. Here we explore to what extent the first prototype of this device complies with these requirements and whether NEX4EX should be developed further into hardware suitable for space travel that enables adequate countermeasure training for maintaining crew health and mission success.

## 2 METHODS

### 2.1 Participants

The study received ethical approval from the Ärztekammer Nordrhein (no. 20213636) before study commencement, and it was implemented at the German Aerospace Center (DLR) Institute of Aerospace Medicine between February and April 2023. The study complied with the *Declaration of Helsinki* and all participants gave written informed consent before the start of the experiments.

We included 11 participants (six females, five males), with mean age of 39 (SD 7) years, mean body mass of 74.6 (SD 12.8) kg and mean height of 175.9 (SD 10.4) cm. All participants were recreationally active.

After finishing the measurement of eight participants, one of the two constant force mechanisms (CFM), which were responsible for applying the load during RES and PLYO, malfunctioned and during repair it was further identified that on the other side a rope jumped off a pulley at the second CFM resulting in increased internal friction. After the repair of both CFMs, the remaining three participants were tested without malfunction. It was decided, therefore, to analyse RES and PLYO data only for the last three participants (two male, one female), since the project schedule did not allow re‐inviting the participants.

### 2.2 NEX4EX device

**FIGURE 1:**
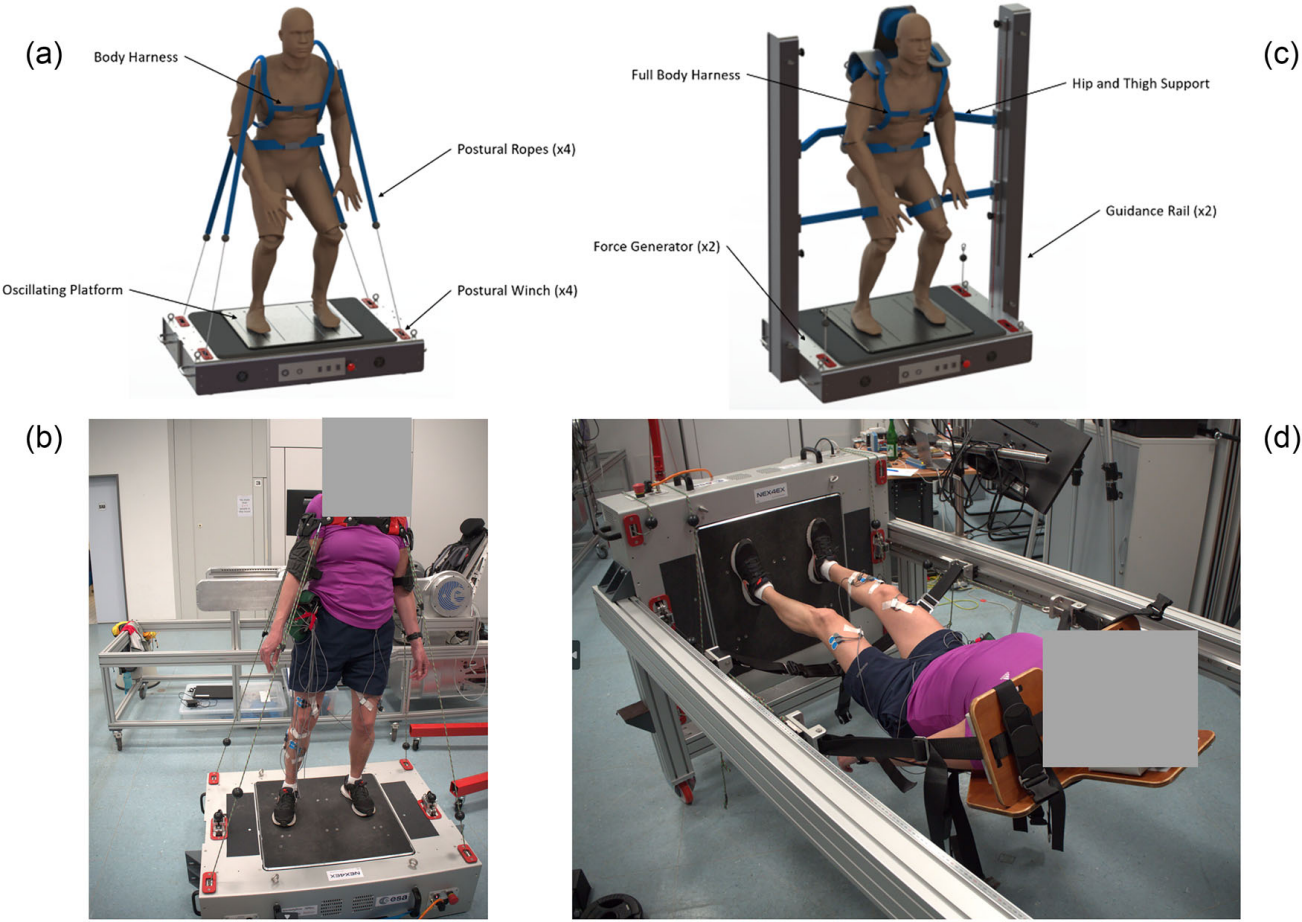
The two different configurations of NEX4EX. (a) Computer rendering of configuration 1 for sensorimotor training including description of the postural winches, the postural ropes and the oscillating platform. (b) Picture of one participant performing the sensorimotor training during the clinical test at the German Aerospace Center. (c) Computer rendering of configuration 2, which is used during resistive and plyometric exercising. Description of guidance rails, force generators, full body harness and hip and thigh support. (d) Mechanical ground support equipment (MGSE) including the jumping sledge and full body harness during the clinical tests at the German Aerospace Center.

The first prototype of the device was designed, built and validated under ESA contract AO/1‐9369/18/NL/KML as an integrated platform for RES, PLYO and SENSO (Figure 1 and Supporting information Figure S1). Its details are described elsewhere (Fau et al., 2022). The validation strategy included recordings (1 kHz sampling rate) of ground reaction force (GRF), loading by the CFMs, sledge displacement, and during SENSO, force and length displacements of the four ropes that were attached to a harness at the shoulders of the participants. A trigger signal was used to synchronise the data provided by NEX4EX and surface‐electromyography (sEMG) data.

### 2.3 Data acquisition

sEMG recordings were obtained (Noraxon Telemyo 2400TG2‐USB, Noraxon, Scottsdale, AZ, USA) from the right tibialis anterior muscle (TA), right gastrocnemius lateralis muscle (GL), right biceps femoris muscle (BF), left and right vastus lateralis muscle (VL_l, VL_r), right rectus abdominis muscle (RA), right erector spinae muscle (ES) at level L3, and right and left obliquus externus abdominis muscles (OEA_r, OEA_l). Signals were sampled at 1.5 kHz, using the synchronisation signal from the NEX4EX device. Furthermore, a three‐dimensional accelerometer was placed underneath the right patella, and data were again sampled with 1.5 kHz in *x*, *y*, *z* directions.

### 2.4 Intervention

We aimed to compare countermovement jumping (CMJ, three valid trials), reactive hopping (HOP, three sets of 20 hops each), squats (SQ) and heel raises (HEEL) between a reference task and the NEX4EX exercises. During reference, all tasks were performed while standing on the ground, whereas during exercising on NEX4EX these same tasks were performed whilst lying on a horizontal sledge to unload the body in the length axis from gravitational force. For CMJ and HOP under reference conditions, participants performed the jumps on a Leonardo platform (Novotec Medical GmbH, Pforzheim, Germany). For SQ and HEEL, participants performed the exercises with a power rack adding 50% of body weight (BW) via a barbell on the shoulders (30 repetitions, or up to failure).

On NEX4EX, RES and PLYO were performed supine using the NEX4EX mechanical ground system equipment (see Supporting information Video S3 (Squats)/Video S5 (Heel_raise)/Video S4 (Countermovement_Jump)/Video S6 (Reactive_Hopping)). Whilst lying on the sledge, the body was loaded by forces generated by a pair of CFMs and transmitted via ropes to a torso‐harness that was suspended in a sledge connected to the mechanical ground support equipment. As under reference conditions, 150% BW was intended to be added for SQ and HEEL. The NEX4EX prototype version was limited at 785 N, and therefore, two out of three participants were loaded lower than the intended 150% BW (94% BW and 105% BW). Force adjustments were conducted with participants flexing their knees to prevent any injuries while increasing the load. In case participants were unable to extend the legs at a given harness load, then the forces were gradually reduced.

For the subsequent PLYO, constant force levels were set to either 100% BW or the value used for RES, whichever was lower. When participants were unable to perform a CMJ, loads were again gradually reduced.

SENSO on NEX4EX comprised disturbances to the upright posture by shoulder pulls or by platform oscillation (Figure 1, Supporting information Video S1 (Sensorimotor_01)/Video S2 (Sensorimotor_02)). For both, participants were loaded with 160 N in total (40 N per motor) to tighten the ropes that connected the electric motors to the shoulder harness. Shoulder pulls were provided by sudden force enhancement by 160–180 N over 500 ms, applied from one single rope, and with 4 s‐breaks between pulls. The resulting pull direction (posterior_left, posterior_right, anterior_left, anterior_right) was randomly varied throughout the 40 pulls applied. Participants were asked to resist the pull in upright posture and to re‐position as quickly as possible. Platform oscillations were provided at four different frequencies (1, 2, 5 and 10 Hz) to the participants, who were asked to maintain upright posture.

Supporting information Table S1 gives a detailed overview of the testing schedule.

### 2.5 Data processing

Data files exported from NEX4EX and from the Noraxon software were merged, synchronised and analysed using the R‐environment (www.r‐project.org) in its versions 4.3.2 and 2023.12.0 for R and RStudio, respectively. Data curation involved discarding SENSO trials without properly performed rope pulls or with sEMG artefacts.

For RES and PLYO, we focused on the main muscular actuators during the specific exercise. The sEMG signals were converted into root mean square values (RMS‐EMG) by rectification and by application of a moving average over 501 sample points (0.334 s). For HOP, the number of sample points was reduced to 201 (0.134 s) to prevent excessive data smoothing as these movements were very fast. The mean maxima RMS‐EMG during reference exercising were used as reference values, which were used for normalising EMG values of NEX4EX exercising and calculation of the relative activation related to reference exercising (%RE). For RES and PLYO customized R scripts were used for calculation of the mean maximum lift, the mean peak GRFs (RES) as well as maximum forces, maximum velocities, jumping heights and mean ground contact times (PLYO).

For SENSO processing, shoulder pulls were identified by segmenting rope force increases, and then we averaged sEMG signals for individual muscles (Supporting information Figures S2–S5). From the averaged signals, we determined sEMG latencies for each muscle in each condition.

As no reliable sEMG responses were found for platform oscillations at 1 and 2 Hz, we discarded those trials and focused on sEMG signals for cycles of 5 Hz and 10 Hz oscillations. Oscillation cycle was identified as nadirs in the vertical acceleration signal (Supporting information Figures S6 and S7).

## 3 RESULTS

### 3.1 Resistive exercise

Whilst both male participants performed RES with the maximum possible force of 785 N on NEX4EX (corresponding to 63% and 70%RE), the force had to be reduced to 589 N for the female participant in order to allow RES in a natural movement pattern (73%RE) (Table 1).

**TABLE 1:** Results of exercise performances on the NEX4EX device in comparison with a reference exercise.

Exercise	Participant	Sex	Given constant force (kN)	Given constant force (%RE)	Lift (cm)	Peak force (kN)	Peak force (%RE)	*V*_max_ (m/s)	*V*_max_ (%RE)	Ground contact time (s)	Ground contact time (%RE)	Jump height (cm)	Jump height (%RE)	VL_r (%RE)	VL_l (%RE)	GL (%RE)
Squatting	1	Male	0.78	63	43	0.97	—	—	—	—	—	—	—	83	84	—
	2	Female	0.59	73	22	0.86	—	—	—	—	—	—	—	71	82	—
	3	Male	0.78	70	36	0.93	—	—	—	—	—	—	—	61	64	—
Heel raises	1	Male	0.78	63	4	1.06	—	—	—	—	—	—	—	—	—	97
	2	Female	0.59	73	4	0.84	—	—	—	—	—	—	—	—	—	108
	3	Male	0.78	70	4	0.96	—	—	—	—	—	—	—	—	—	76
Countermovement Jumps	1	Male	—	92	—	1.36	83	0.87	36	—	—	11	28	197	179	112
	2	Female	—	76	—	0.97	83	0.71	34	—	—	13	42	56	64	83
	3	Male	—	100	—	1.93	104	1.31	47	—	—	14	30	89	89	60
Reactive Hopping	1	Male	—	92	—	1.54	38	—	—	0.16	80*	6	38	120	95	79
	2	Female	—	76	—	1.09	54	—	—	0.38	200	5	43	99	99	134
	3	Male	—	100	—	2.24	58	—	—	0.17	113	6	45	76	84	101

During reference squatting and reference heel raises using a barbell with 50% body weight extra load. RE: reference exercise. Peak force is before the flight phase. *V*_max_: maximum velocity before flight phase. Ground contact time: mean ground contact time between the reactive forefoot hops. Mean of EMG‐RMS peak amplitudes from vastus lateralis (VL) and gastrocnemius lateralis (GL) muscles, r: right, l: left. *Participant performed a lift‐off for every second hop, and thus the forefoot maintained at the platform for the hops between. This resulted in an improved ground contact time, although it was no real hopping exercise. As we had to exclude most data sets from analysis, we decided to keep this data set as it shows that reactive hopping is in principle possible.

The peak muscle activation in the vastus lateralis muscle was reduced on average in the same range as the load. Despite the reduced loads, the muscle activation during HEEL was comparable for both conditions. A lift of 4 cm was measured during HEEL.

### 3.2 Plyometric exercise

The applied load during PLYO ranged from 76%RE to 100%RE. The exercises were performed with a reduced maximum velocity (34%RE–47%RE) resulting in a reduced jumping height (28%RE–42%RE). The activation level of GL ranged from 60%RE to 112%RE. For CMJ, the VL_r showed activation levels from 56%RE to 197%RE, VL_l from 64%RE to 179%RE (Table 1).

**FIGURE 2:**
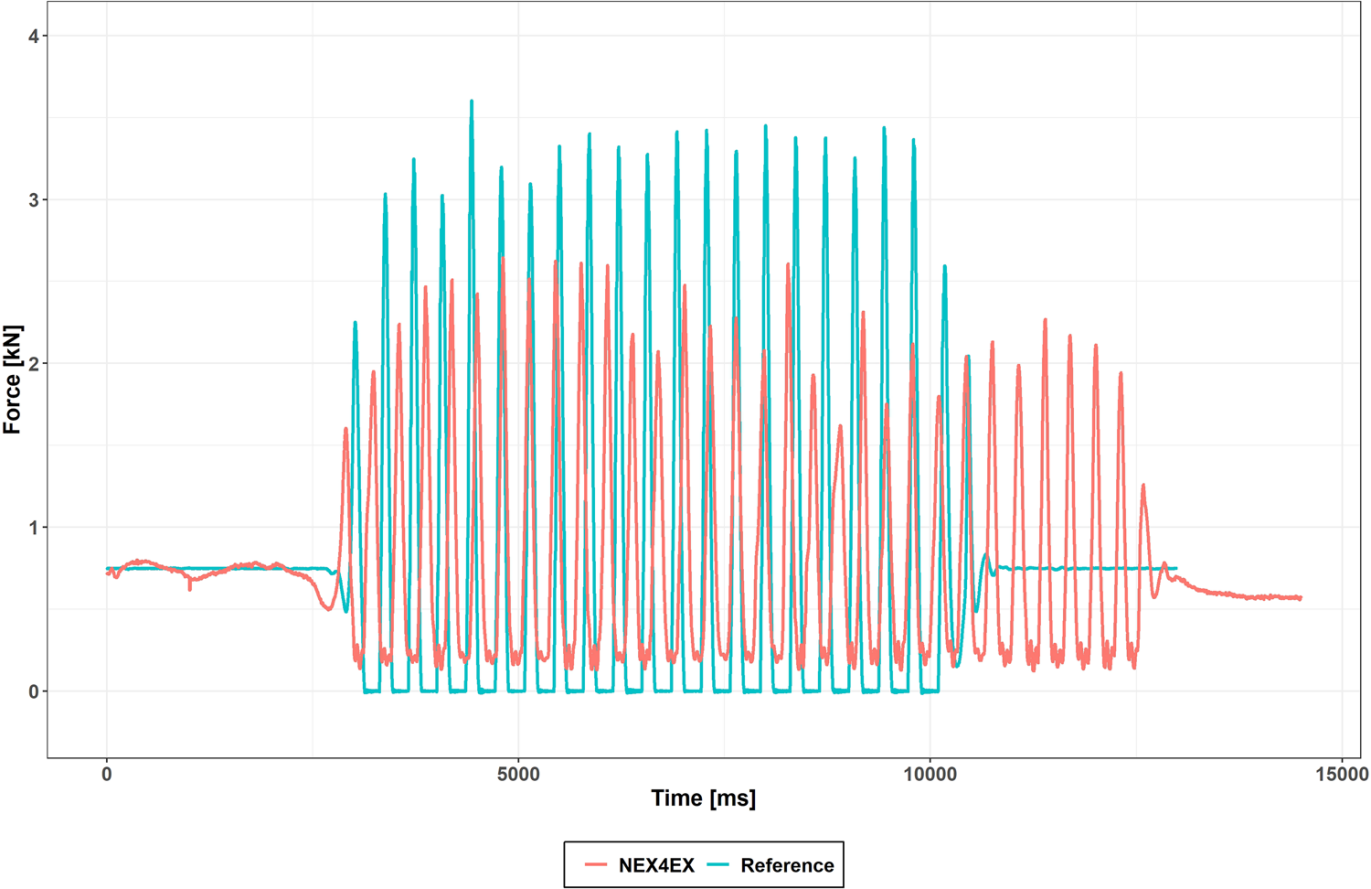
The ground reaction force (GRF) during reference hopping in vertical direction on a jumping platform (turquoise) and during hopping on NEX4EX in horizontal direction (red). As the NEX4EX device did not enable a zero adjustment, there was an offset. Furthermore, the unloaded NEX4EX platform showed vibrations during the plyometric exercises. These data are from one participant, who performed the reactive hopping well under both conditions. Both curves showed single peaks per hop and the ground contact times are comparable. The total time for hopping on NEX4EX is just longer because more hops were performed in this set.

HOP was performed with the same applied load as CMJ, but the resulting peak force was lower. If compared to the reference hopping, the peak force ranged from 38%RE to 58%RE. The ground contact time lasted between 0.16 and 0.38 s (80%–200%RE), whereas one participant showed a lift‐off just for every second HOP (Figure 2). GL showed an activation level from 79%RE to 134%RE, VL_r from 76% to 120%RE and VL_l from 84% to 99%RE (Table 1).

### 3.3 Sensorimotor exercise

The four different perturbation stimuli caused specific neuromuscular reactions of lower extremity and trunk muscles (Supporting information Figures S2–S5). The time delay of onset of muscle activity after the perturbation stimulus was in the range of 80–110 ms for most muscles after specific stimulation configurations (Table 2). Such latencies indicate the existence of primarily medium and late reflex responses (Taube et al., 2008). However, when correcting for delays between stimulation at the shoulders and start of body movement, which was measured by acceleration of the shank, muscle response latencies ranged between 25 and 33 ms in TA and some trunk muscles (RA during posterior_left pull, ES during posterior_right pulls, OEA_r during posterior_left and anterior_left pulls), whilst latencies of the other muscles were 30–40 ms. Such latencies correspond well with short latency reflexes of the fastest spinal reflex contributions (Taube et al., 2008) that contribute to the stabilisation of body posture (see Table 2). Oscillating platform stimulation at the level of feet elicited distinct responses in lower extremity and trunk muscles at 10 Hz only, and only with substantially smaller response amplitudes than those observed in response to shoulder pulls (see Supporting information Figures S6 and S7).

**TABLE 2:** Mean latencies of muscle activity as a reaction towards the postural perturbation by the rope pulls.

	Pull posterior_left		Pull posterior_right		Pull anterior_left		Pull anterior_right	
	Latency pull (ms)	Latency acc (ms)	Latency pull (ms)	Latency acc (ms)	Latency pull (ms)	Latency acc (ms)	Latency pull (ms)	Latency acc (ms)
Tibialis anterior	85	25–32	85	25–32	100	40–47	101	41–48
Gastrocnemius lat.	No	No	99	39–46	95	35–42	101	41–48
Biceps femoris	90	30–37	107	47–55	106	46–53	114	54–61
Vastus lateralis	94	34–41	87	27–34	181	121–128	142	82–89
Vastus lateralis (left)	98	38–45	113	53–60	120	60–67	149	89–96
Erector spinae	99	39–46	85	25–32	94	34–41	119	59–66
Rectus abdominis	84	24–31	98	38–45	91	31–38	124	64–71
Obliquus externus abdominis	85	25–32	88	28–35	85	25–32	91	31–38
Obliquus externus abdominis (left)	89	29–36	87	27–34	88	28–35	93	33–40

All four conditions were analysed (posterior_left, posterior_right, anterior_left, anterior_right). Two latencies were calculated, the latency for the pull force and for the acceleration signal measured at the right shank. The following muscles were assessed: right tibialis anterior muscle as dorsal flexor; right gastrocnemius lateralis muscle representing the plantarflexor muscles; right biceps femoris muscle as knee flexor; left and right vastus lateralis as knee extensors; right rectus abdominis muscle as flexor of the trunk, right erector spinae muscle (level L3) as extensor of the trunk, and right and left obliquus externus abdominis muscles as rotational muscles of the trunk.

## 4 DISCUSSION

### 4.1 Resistive exercises

The applied load of 150% body weight during reference exercise allowed all participants to perform all kinds of resistive exercise with 20 or more repetitions, which indicates a low to moderate load. Unfortunately, the dimensions of NEX4EX enabled just a maximum force of 785 N, which is equal to the weight of 80 kg body mass. It can be stated that SQ with reduced loads elicited muscle activation levels, which were reduced about the same amount. Thus, one can assume that squats performed on NEX4EX with 150% body weight would likely cause comparable muscle activation as during reference exercise.

For HEEL on NEX4EX, a lift of about 10 cm was expected due to the foot lengths, but a maximum lift of only 4 cm was measured. The design of the sledge carrying the participants enabled a swing, which falsified the true lift as it was measured by the length change of the ropes connecting the force generators with the sledge.

### 4.2 Plyometric exercises

For PLYO, the three participants showed a comparable muscle activity with a lower applied load. Of course, these are only initial tendencies without statistical evidence, but these tendencies were clearly recognizable in the muscle groups that are essential for performing the respective exercise (VL_r and VL_l during CMJ; GL during HOP). Furthermore, two participants reached or even improved reference ground contact time during HOP, but one of the two participants did really lift‐off every second hop. It follows that an adequate training process could enable all participants to perform HOP with comparable ground contact times as stated by Kramer et al. (2018).

### 4.3 Sensorimotor exercise

The perturbations at the shoulders elicited distinct activation pattern of shank, thigh and trunk muscles, whereas the vibration did not elicit a clear muscle response. The muscle activity latencies indicated that spinal reflexes were triggered by the stimulus. This is of importance as many of the stabilizing muscles of the trunk play a major role in postural control, located deep in the abdomen, pelvis and back. We did not measure activity patterns of deep trunk stabilizers; however, the reflex activity that we observed in superficial muscles indicates that using NEX4EX possibly provides an adequate training stimulus for deep muscles that are mainly under spinal reflex control (Ivanenko & Gurfinkel, 2018). As a future development, the perturbating stimuli could be designed as a random combination of multiple rope pulls at the same time eliciting further muscle activity. Another modification would be to tailor the training by variations of the force intensity applied over the rope to the human body. A third possibility would be to change the stance of the trained person.

### 4.4 Limitations

As this is a case report, the results only indicate tendencies. These need to be verified with a larger number of participants. Nevertheless, these initial tendencies are promising. On this prototype of the device, the participants could not perform maximum voluntary contraction for normalisation of the sEMG signal. Therefore, we used the reference measurements for the normalisation process, which was adequate from our point of view. Furthermore, we did not perform any biomechanical analysis regarding a comparable movement between reference and NEX4EX exercising, which should be performed in future experiments as the sledge, for example, has a direct influence on the movement execution.

### 4.5 Conclusions

All intended exercises were feasible using NEX4EX. Resistive exercises and plyometric exercises were possible with reduced loads; sensorimotor exercises evoked postural reflexes of the trunk and leg muscles. The device demonstrated great potential for being an adequate countermeasure device for deep‐space missions. However, to enable a sufficient loading of all participants during resistive and plyometric exercises, the maximal capacity of the CFM needs to be doubled.

### 4.6 Participant perspective

Most participants gave feedback during or after the measurement, with most of the feedback relating to the sledge. It was noted that the sledge had a left skew, that it becomes uncomfortable on the shoulders after a long time with high loads, that the movement does not feel natural and that the sledge was too small for tall participants, whereas it was too large for small people. Thus, the next iteration of NEX4EX should focus on redesigning the sledge to improve the comfort of the participants and prevent any unilateral shifting. For SENSO, it was noted that the harness became uncomfortable as the hands fell asleep. At the start of the postural disturbance exercise, the impact of the first pull felt as a surprise by the participants, and hence could increase the risk of falling and, thus, injuries.

## AUTHOR CONTRIBUTIONS

Jonas Böcker, Jochen Zange, Markus Gruber and Jörn Rittweger contributed to the design and implementation of the study. Guillaume Fau and Rainer Rawer developed the countermeasure device. Jonas Böcker, Jochen Zange and Markus Gruber conducted the tests and evaluated the data. Jonas Böcker, Jochen Zange, Markus Gruber, Guillaume Fau and Rainer Rawer wrote the manuscript. All authors revised the manuscript. All authors have read and approved the final version of this manuscript and agree to be accountable for all aspects of the work in ensuring that questions related to the accuracy or integrity of any part of the work are appropriately investigated and resolved. All persons designated as authors qualify for authorship, and all those who qualify for authorship are listed.

## Supporting information



Supplementary Table 1 and Supplementary Figures 1–7.

Video S1. Sensorimotor 01

Video S2. Sensorimotor 02

Video S3. Squats

Video S4. Countermovement Jump

Video S5. Heel raise

Video S6. Reactive Hopping

## Data Availability

The data are stored locally at the German Aerospace Center. The data can be made available on request with appropriate justification and approval by ESA.
