# Supplementary material for: NEX4EX – A novel exercise device enabling resistive, plyometric and sensorimotor training during deep‐space missions: A case report
Source: Exp Physiol. 2025 Sep 15;111(9):3936–43. doi: 10.1113/EP092721 (PMC13394085; doi:10.1113/EP092721)
Supplement: Supplementary file 1 [file EPH-111-3936-s005.pdf]

Supplementary Material

Supplementary Table 1: Schedule of testing per participant. Steps 8-11 and 12-15 were changed randomly. The total duration of each measurement was about 160 minutes.

Step No.	Duration [min]	Task
1	30	Measurement preparation: Fixing electrodes of electromyography (EMG) system (lower and upper legs, hips, trunk covering all set-ups) and electro cardiogram (ECG) electrodes to the participant
2	5	General warm-up: submaximal countermovement jumps, hopping
3	5	Plyometric exercising: Repeated Countermovement Jumps (Force Plate/Jumping Platform)
4	5	Plyometric exercising: Reactive hopping (Force Plate/Jumping Platform)
5	5	General warm-up: submaximal squats and heel raises
6	10	Low to medium resistive training: Squats (Smith machine) with 50% body weight (BW) as additional load
7	10	Low to medium resistive training: Heel raises (Smith machine) with 50% BW as additional load
8	10	Donning configuration 1 (upright, vertical position of the participant): Harness setup, fitting the harness to the participant, positioning participant to the device, fixing the 4 ropes
9	10	Postural control training with disturbances at the shoulders
10	10	Postural control training with low frequency oscillation at the feet
11	15	Removing configuration 1; Donning configuration 2 (lying, horizontal position of the participant)
12	10	Resistive exercising: Squats
13	10	Resistive exercising: Heel raises
14	10	Plyometric exercising: Repeated countermovement jumps
15	5	Plyometric exercising: Reactive hopping

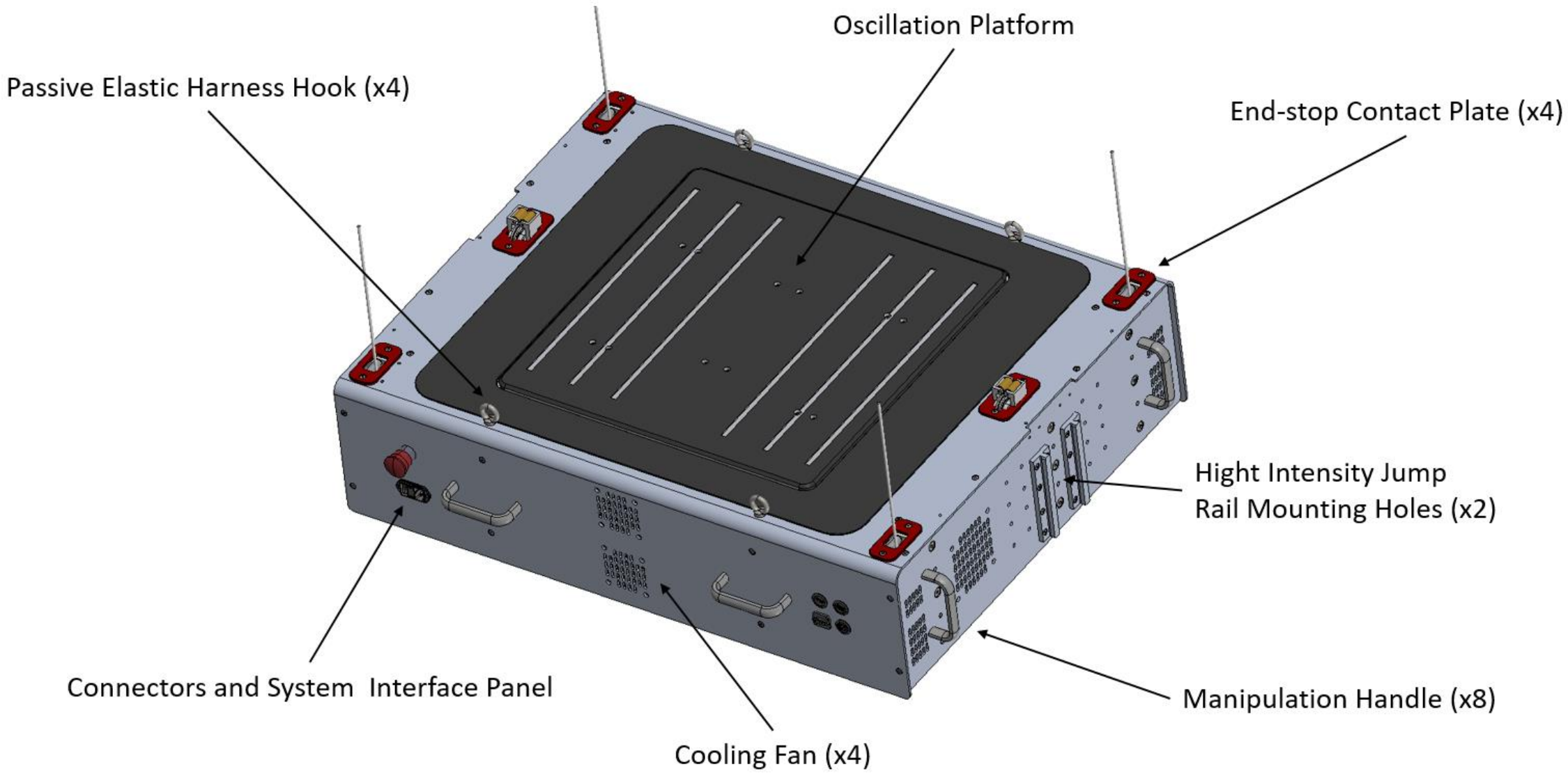

Supplementary Figure 1: The NEX4EX platform including sub-modules for four postural stimulation motors (in the four corners including End-Stop Contact Plate), an oscillation ground-reaction platform and two passive artificial gravity generators (constant force mechanisms) as well as all the onboard control electronics and computer units.

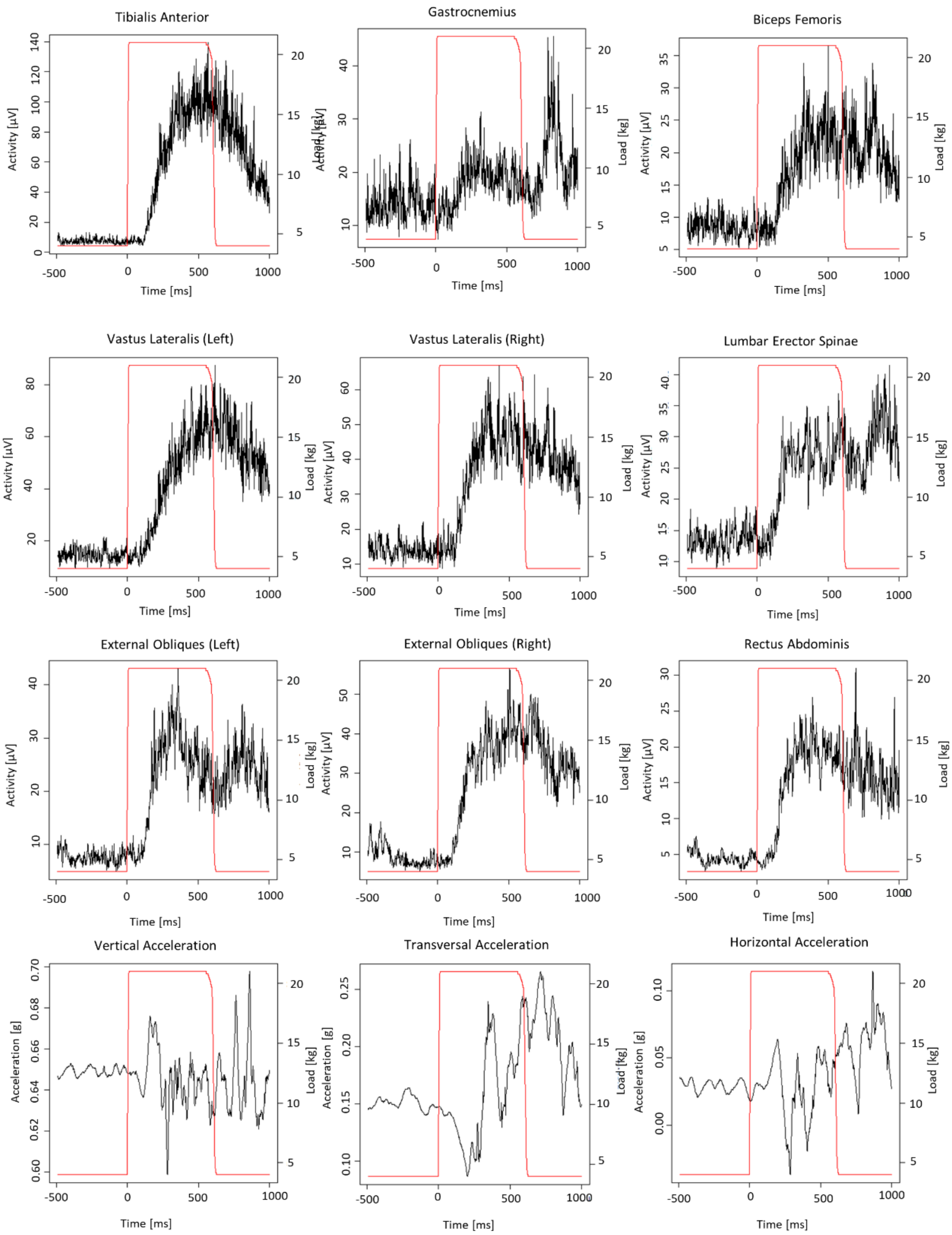

Supplementary Figure 2: Muscle activities (black, rows 1-3) and acceleration (grey, row 4) 500ms before and 1000ms after the rope pull (posterior_left) and the resulting pull-forces (red). The individual trials were averaged individually and then averaged for the whole group. The rope pulled the participant back to the left side in their stance and caused a reactive response in all recorded muscles. There are clear onsets of muscle activity after the perturbation (see latencies in Table 1)

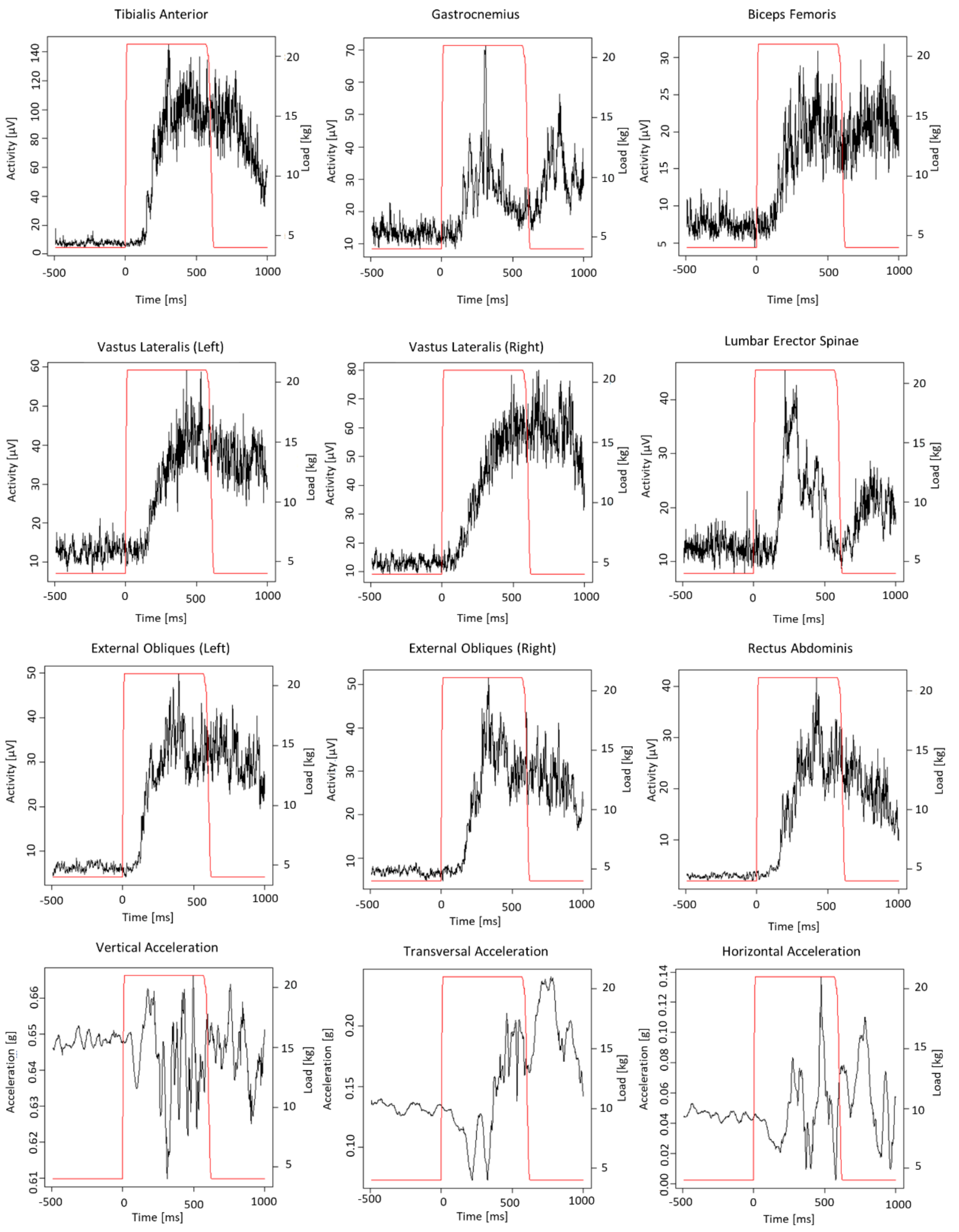

Supplementary Figure 3: Muscle activities (black, rows 1-3) and acceleration (grey, row 4) 500ms before and 1000ms after the rope pull (posterior_right) and the resulting pull-forces (red). The individual trials were averaged individually and then averaged for the whole group. The rope pulled the participant back to the right side in their stance and caused a reactive response in all recorded muscles. There are clear onsets of muscle activity after the perturbation (see latencies in Table 1)

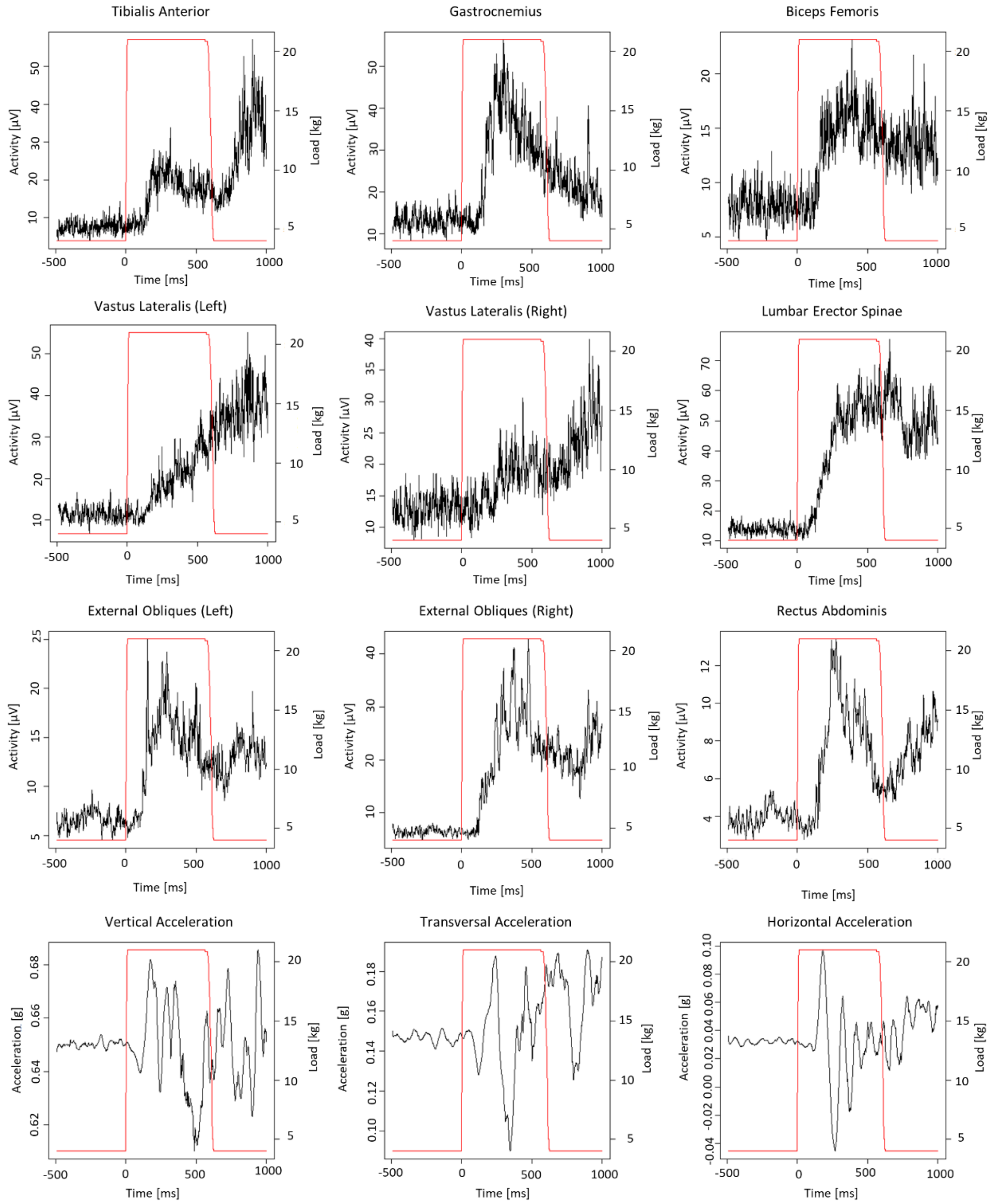

Supplementary Figure 4: Muscle activities (black, rows 1-3) and acceleration (grey, row 4) 500ms before and 1000ms after the rope pull (anterior_left) and the resulting pull-forces (red). The individual trials were averaged individually and then averaged for the whole group. The rope pulled the participant forward to the left side in their stance and caused a reactive response in all recorded muscles. There are clear onsets of muscle activity after the perturbation (see latencies in Table 1)

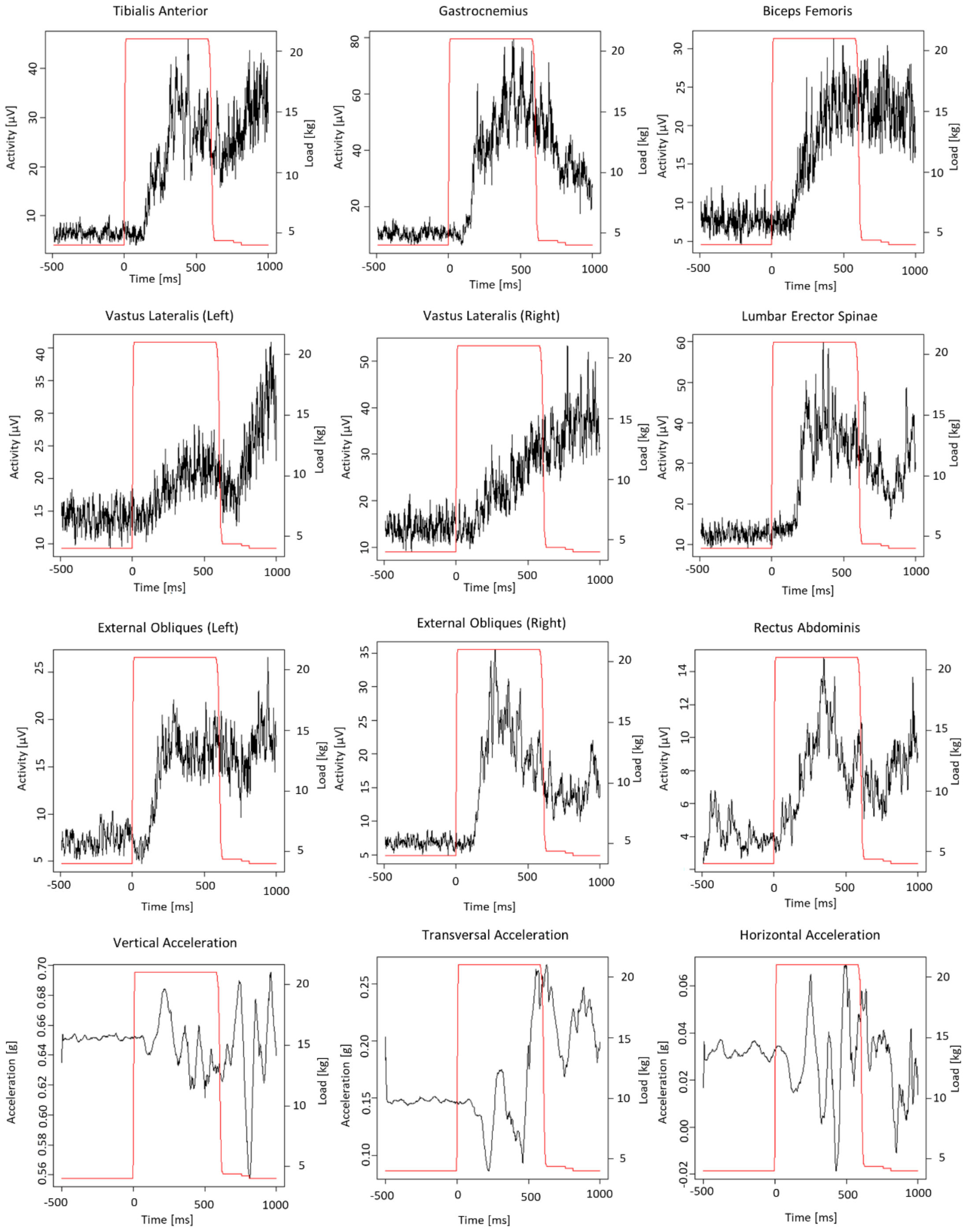

Supplementary Figure 5: Muscle activities (black, rows 1-3) and acceleration (grey, row 4) 500ms before and 1000ms after the rope pull (anterior_right) and the resulting pull-forces (red). The individual trials were averaged individually and then averaged for the whole group. The rope pulled the participant forward to the right side in their stance and caused a reactive response in all recorded muscles. There are clear onsets of muscle activity after the perturbation (see latencies in Table 1)

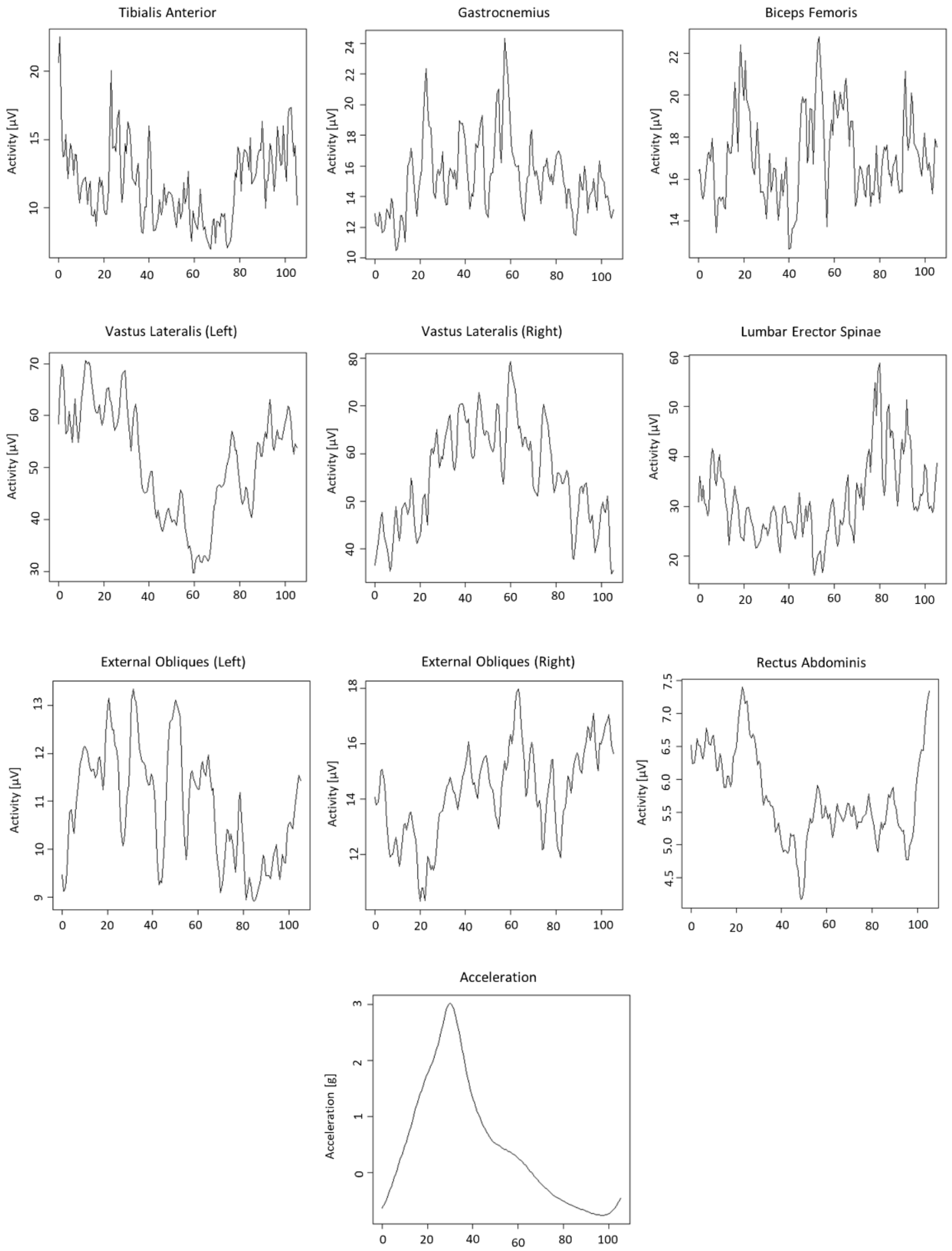

Supplementary Figure 6: Activity patterns of the recorded muscles during the vibration stimulus (≈10 Hz; ≈100ms). The muscle activity was averaged individually for 10 cycles and then averaged for the group. The pattern shows the antagonistic function of TA and GL, as well as the synergistic function of GL and BF. However, it should be noted that activity levels are in general low. A phase specific behavior of VL_l vs. VL_r can be observed which is most probably related to the alternating vibration and the pressure shift from the right to the left leg and back. Trunk muscle activity is rather low with the exception of ES, that shows a considerable increase in muscle activity during the second half of the vibration cycle.

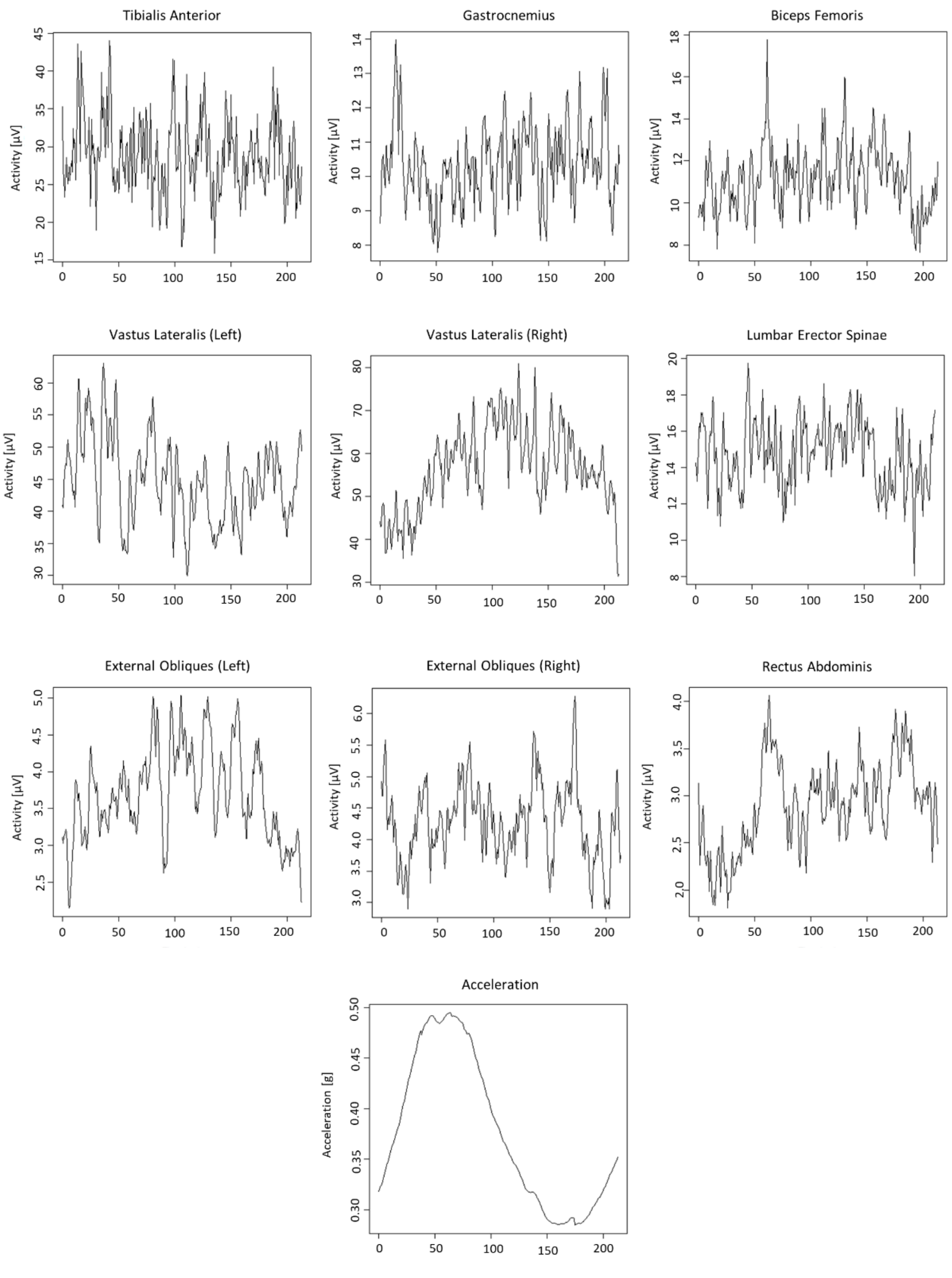

Supplementary Figure 7: Activity patterns of the recorded muscles during the vibration stimulus (≈5 Hz, ≈200 ms). The muscle activity was averaged individually for 10 cycles and then averaged for the group. There is no clear activity pattern of the individual muscles. The only pattern that is left is a just indicated phase specific behaviour of VL_l vs. VL_r which, like we argued for the Hz frequency, is most probably related to the alternating vibration and the pressure shift from the right to the left leg and back.
